# COVID-19 and Maternal Death in Brazil: An Invisible Tragedy

**DOI:** 10.1055/s-0040-1715138

**Published:** 2020-09-08

**Authors:** Marcos Nakamura-Pereira, Melania Maria Ramos Amorim, Rodolfo de Carvalho Pacagnella, Maira Libertad Soligo Takemoto, Fatima Cristina Cunha Penso, Jorge de Rezende-Filho, Maria do Carmo Leal

**Affiliations:** 1 Instituto Nacional de Saúde da Mulher, da Criança e do Adolescente Fernandes Figueira, Fundação Oswaldo Cruz, Rio de Janeiro, RJ, Brazil; 2 Instituto de Medicina Integral Prof. Fernando Figueira, Recife, PE, Brazil; 3 Universidade Estadual de Campinas, Campinas, SP, Brazil; 4 Faculdade de Medicina de Botucatu, Universidade do Estado de São Paulo, Botucatu, SP, Brazil; 5 Maternidade Leila Diniz, Hospital Municipal Lourenço, Rio de Janeiro, RJ, Brazil; 6 Universidade Federal do Rio de Janeiro, Rio de Janeiro, RJ, Brazil; 7 Escola Nacional de Saúde Pública Sergio Arouca, Fundação Oswaldo Cruz, Manguinhos, RJ, Brazil

The infection with the new severe acute respiratory syndrome coronavirus 2 (SARS-CoV-2), which is responsible for causing the coronavirus disease 2019 (COVID-19), became a devastating threat to the health of the world population and was declared a global pandemic by the World Health Organization (WHO) on March 11, 2020. Beginning in China at the end of 2019, it quickly spread to several countries, and the first case was officially diagnosed in Brazil on February 26, 2020. Since then, despite initial measures to slow the virus' spread, we are alarmed by the exponential growth in the number of cases. At the time of writing this text, Brazil exceeds 800,000 cases and 40,000 deaths, second only to the United States in those numbers. However, the well-known underreporting of cases and deaths in the country, associated with the incomprehensible decision to suspend access to the Epidemiological Surveillance Information System - Influenza (SIVEP-Gripe, in the Portuguese acronym) database for recalculating the number of deaths makes it difficult to keep these numbers up to date.

The real impact of COVID-19 on pregnancy, childbirth and the puerperium period, and if the pregnancy-puerperal state changes the natural history of COVID-19 are controversial issues that remain to be elucidated. Initial studies in the obstetric population were not suggestive of a greater susceptibility of pregnant women to COVID-19 complications. 1 2 However, subsequent publications reported cases of pregnant women with severe disease and maternal deaths from COVID-19. 3 4 5 6 7 8 A Swedish study showed a higher risk of pregnant women needing admission to the intensive care unit compared with non-pregnant women. 7 To date, reports of maternal death have been published in Iran and Mexico (seven cases each), in the United Kingdom (five deaths) and in the United States (one case). 4 5 6 8

These data raise concerns about the impact of COVID-19 on maternal mortality in Brazil. In April 2020, the Brazilian Ministry of Health included all pregnant and puerperal women and patients with gestational or fetal loss up to day 15 in the risk group for COVID-19. However, until the end of May there was no official release of aggregated death data during pregnancy and postpartum. Amorim et al (2020), 9 using official and media data, have preliminarily reported 5 cases of maternal death in the country until April 10, 2020. Finally, at the end of May, the Ministry of Health 10 reported the occurrence of 36 cases of maternal death from COVID-19 and 252 cases of severe acute respiratory syndrome (SARS) occurring up to epidemiological week 21 (May 17–23, 2020). 10

The identification of these 36 deaths already places Brazil in the leadership of maternal deaths due to COVID-19 in the world, even when adding all maternal deaths published in the literature so far. What would justify so many deaths from COVID-19 in our country? Is it possible that the population of pregnant women has very different characteristics from pregnant women in other countries, and that the presence of comorbidities, especially preeclampsia and obesity, which are very common inflammatory conditions in our population and risk factors for complications due to COVID-19, can explain these findings? 11 12 13 Hypertension is the leading cause of maternal death and maternal near-miss in Brazil, and there is evidence of a similar picture of preeclampsia observed in women with severe COVID. 14 15 Data from the Ministry of Health show a higher incidence of hypertension among pregnant women and women who have recently died, compared with those who have had SARS and recovered (13.9% versus 3.9%). 10 Thus, when hypertension and COVID-19 infection occur simultaneously in pregnancy, the inflammatory response may contribute to a worse maternal prognosis. Obesity (11.1% versus 4.4%) is another condition of high incidence among fatal cases in Brazil. 10 Brazil has a high prevalence of overweight and obese pregnant women, 16 and the metabolic syndrome is also a proinflammatory state, just like the systemic response observed in severe cases of COVID. In France, obesity is a risk factor for severe maternal morbidity and there is no reason to suppose that such an association is not present in Brazil. 13 In addition, obesity may contribute to maternal death because of its association with preeclampsia. 12

In Brazil, barriers to access services with specialized care and inadequate monitoring of obstetric complications also persist in hospitals, primary care and specialty clinics, as evidenced in two large national studies. 17 18 In addition, there are structural deficiencies in Brazilian maternity hospitals, both in physical and in human and material resources (medicines, laboratory, etc.). In the public system, only 15% of maternity units have an adult intensive care unit (ICU), and the availability of places is extremely uneven across the Brazilian territory. 19 Consequently, maternal deaths in Brazil are more related to phases II and III of delays 17 and this was possibly intensified in this pandemic period. In general, for the better management of cases of symptomatic people during the pandemic period, Basic Health Units have postponed prenatal consultations, which has increased the barriers to adequate prenatal care. As a result of this approach, pregnant women have arrived in hospitals in more serious clinical conditions that could have been avoided with timely and quality prenatal care.

These deficiencies in the health system, potentially aggravated by the recent economic crisis and restrictions of public investments in health, are put in check at this moment because all resources and attention are focused on care for the COVID-19 pandemic. Roberton et al, in statistical modeling to assess the indirect effects of COVID-19, estimated that maternal mortality could increase between 8.3 and 38.6% per month in low and middle income countries and that 60% of this effect could result from the reduction of four essential interventions: parenteral administration of uterotonics, antibiotics, anticonvulsants, and a clean environment for childbirth. 20

At this moment, it is difficult to measure the impact of the COVID-19 pandemic on maternal mortality in Brazil. The growing figures and media reports will certainty cause an increase in relation to previous years. Brazil has failed to meet the millennium goal of reducing maternal mortality by 75% from 1990 to 2015, and, since 2012, there has been no sustained reduction in maternal mortality. 21 We are still far from the goal set by the federal government of 30 deaths per 100,000 live births by 2030 to contribute to the WHO Sustainable Development Goals, a plan to which Brazil is a signatory country.

In the current context of the pandemic, it is essential to continue the usual obstetric care with prenatal care in basic units and maternity units by facilitating pregnant women's access to health services, ensuring essential supplies for hospitals with obstetric care and continuing the services of family planning and abortion provided by law. In this particular matter, we observe with perplexity and concern the revocation of Technical Note number 16/2020 (COSMU/CGCIVI/DAPES/SAPES/MS), which only ensured access to sexual and reproductive health within the pandemic context ( https://kidopilabs.com.br/planificasus/upload/covid19_anexo_46.pdf ). Managers should also ensure the rapid identification of pregnant and postpartum women with symptoms suggestive of COVID-19, ideally by offering universal testing 22 23 and making ICU beds available in a timely manner to avoid delays in proper treatment.

Finally, it is necessary to think about the postpandemic moment. Part of the advanced support equipment acquired by federal entities because of COVID-19 can be reverted to our maternity hospitals later. However, for reducing maternal mortality in Brazil, it is necessary to reduce delays in accessing the health system and receiving adequate, respectful, and quality care. 24 Recently, the Brazilian Federation of Gynecology and Obstetrics Associations (FEBRASGO, in the Portuguese acronym) Specialized National Commission on Maternal Mortality has contributed to the debate on the reduction of maternal mortality in Brazil by making several proposals 25 and remains at the disposal of managers to assist in the development of proposals and implementation of actions aimed at improving obstetric care and, consequently, reducing maternal mortality.

The death of a woman in the pregnancy-puerperal cycle is always a tragedy. Maternal death must be considered a preventable tragedy and every effort must be made to prevent it. The lives of pregnant and postpartum women matter!
